# Prevalence of Poor Sleep Quality in Patients With Hypertension in China: A Meta-analysis of Comparative Studies and Epidemiological Surveys

**DOI:** 10.3389/fpsyt.2020.00591

**Published:** 2020-06-30

**Authors:** Lin Li, Lu Li, Jing-Xin Chai, Le Xiao, Chee H. Ng, Gabor S. Ungvari, Yu-Tao Xiang

**Affiliations:** ^1^Department of Pharmacy, The First Affiliated Hospital of Zhejiang University, Hangzhou, China; ^2^Department of Pharmacy, The Affiliated Brain Hospital of Guangzhou Medical University (Guangzhou Huiai Hospital), Guangzhou, China; ^3^Beijing Centers for Disease Prevention and Control, Beijing, China; ^4^Beijing Centers for Disease Preventive Medical Research, Beijing, China; ^5^The National Clinical Research Center for Mental Disorders & Beijing Key Laboratory of Mental Disorders, Beijing Anding Hospital & the Advanced Innovation Center for Human Brain Protection, Capital Medical University, Beijing, China; ^6^Department of Psychiatry, The Melbourne Clinic and St Vincent’s Hospital, University of Melbourne, Richmond, VIC, Australia; ^7^Division of Psychiatry, School of Medicine, University of Western Australia, Perth, WA, Australia; ^8^Department of Psychiatry, University of Notre Dame Australia, Fremantle, WA, Australia; ^9^Unit of Psychiatry, Faculty of Health Sciences, Institute of Translational Medicine, University of Macau, Macau, China; ^10^Center for Cognition and Brain Sciences, University of Macau, Macau, Macau

**Keywords:** poor sleep quality, meta-analysis, hypertension, China, epidemiology

## Abstract

**Objective:**

This meta-analysis examined the prevalence of poor sleep quality and its associated factors in patients with hypertension in China.

**Methods:**

Both English (PubMed, PsycINFO, EMBASE) and Chinese (Wan Fang Database and Chinese National Knowledge Infrastructure) databases were systematically and independently searched. The random-effects model was used to estimate the prevalence of poor sleep quality in Chinese patients with hypertension. The funnel plot and Egger’s tests were used to assess publication bias.

**Results:**

The prevalence of poor sleep quality in 24 studies with 13,920 hypertensive patients was 52.5% (95% confidence interval [CI]: 46.1–58.9%). In contrast, the prevalence of poor sleep quality in six studies with 5,610 healthy control subjects was 32.5% (95% CI: 19.0–49.7%). In these studies, compared to healthy controls, the pooled odds ratio (OR) of poor sleep quality was 2.66 (95% CI: 1.80–3.93) for hypertensive patients. Subgroup and meta-regression analyses revealed that patients in hospitals were more likely to have poor sleep quality than patients in the community. Studies with smaller sample size, studies using convenience or consecutive sampling and those published in Chinese reported higher prevalence of poor sleep quality. Furthermore, poor sleep quality was more common in older and male hypertensive patients, while the proportion of poor sleep quality was negatively associated with survey year.

**Conclusion:**

Appropriate strategies for screening, prevention, and treatment of poor sleep quality in this population should be developed.

## Introduction

Hypertension is a major public health burden and is associated with severe negative health outcomes. The World Health Organization reported that the number of people with raised blood pressure increased from 594 million in 1975 to 1.13 billion in 2015, with the increase mainly occurring in low- and middle-income countries (1). Hypertension-related complications account for approximately 9.4 million deaths worldwide each year, of which around half are due to heart disease and stroke (2, 3). Apart from cardiovascular diseases, other common complications of hypertension include kidney failure, blindness, and cognitive impairment (2).

Symptoms associated with hypertension, such as headache, chest pain, dizziness, shortness of breath, and nose bleeds (2, 4, 5), often lead to poor sleep quality (6). For example, a large-scale population study conducted in China found that patients with hypertension had worse sleep quality than the general population (7). Other studies also found an association between poor sleep quality with hypertension (7–9). Poor sleep quality was associated with increased risk of physical diseases (10, 11), such as obesity (12), and coronary artery disease (13). In addition, poor sleep quality had a bidirectional association with psychiatric disorders (14, 15). For example, persons with poor sleep quality were more likely to develop depression (16) and anxiety (17). In contrast, patients with psychiatric disorders, such as depression and anxiety, were more likely to have poor sleep quality (18, 19). Adequate epidemiological studies of sleep quality in hypertensive patients are important to reduce its negative health consequences and develop appropriate interventions.

The findings of numerous epidemiological surveys of poor sleep quality in hypertensive patients vary greatly, with prevalence ranging from 14.9 to 85.7% globally (20–23). There is growing evidence that socioeconomic and cultural factors may significantly influence sleep patterns and quality (24, 25), therefore sleep quality should be examined separately in different populations. In China the prevalence of hypertension is 29.6%, indicating that there are approximately 325 million patients with hypertension (26). The findings regarding poor sleep quality in Chinese hypertensive patients are inconsistent across studies, which are probably due to different diagnostic tools, study locations, and definitions used. In addition, most studies on prevalence of poor sleep quality in patients with hypertension published in Chinese are generally not accessible to the international readership and have not been included in prior reviews.

To date, no meta-analysis of poor sleep quality in hypertensive patients in China has been reported. Hence, using comparative and epidemiological studies, we conducted a meta-analysis of the pooled prevalence of poor sleep quality in Chinese hypertensive patients and its associated factors. Sleep quality is evaluated either by self-reported or interviewer-rated scales or physiological measures (such as polysomnography and actigraphy) (27). Empirical evidence showed that self-reported measures are user-friendly, reliable, and sensitive to change in sleep patterns and quality (28, 29). Of the different measures on sleep quality, the Pittsburgh sleep quality index (PSQI) is the most widely used, with satisfactory psychometric properties (30, 31). In China, the Chinese-version of PSQI is the only standardized scale on subjective sleep quality available (32). In order to ensure the homogeneity of included studies, this meta-analysis on sleep quality therefore only included studies using the PSQI.

## Methods

### Search Strategies

**Figure 1:**
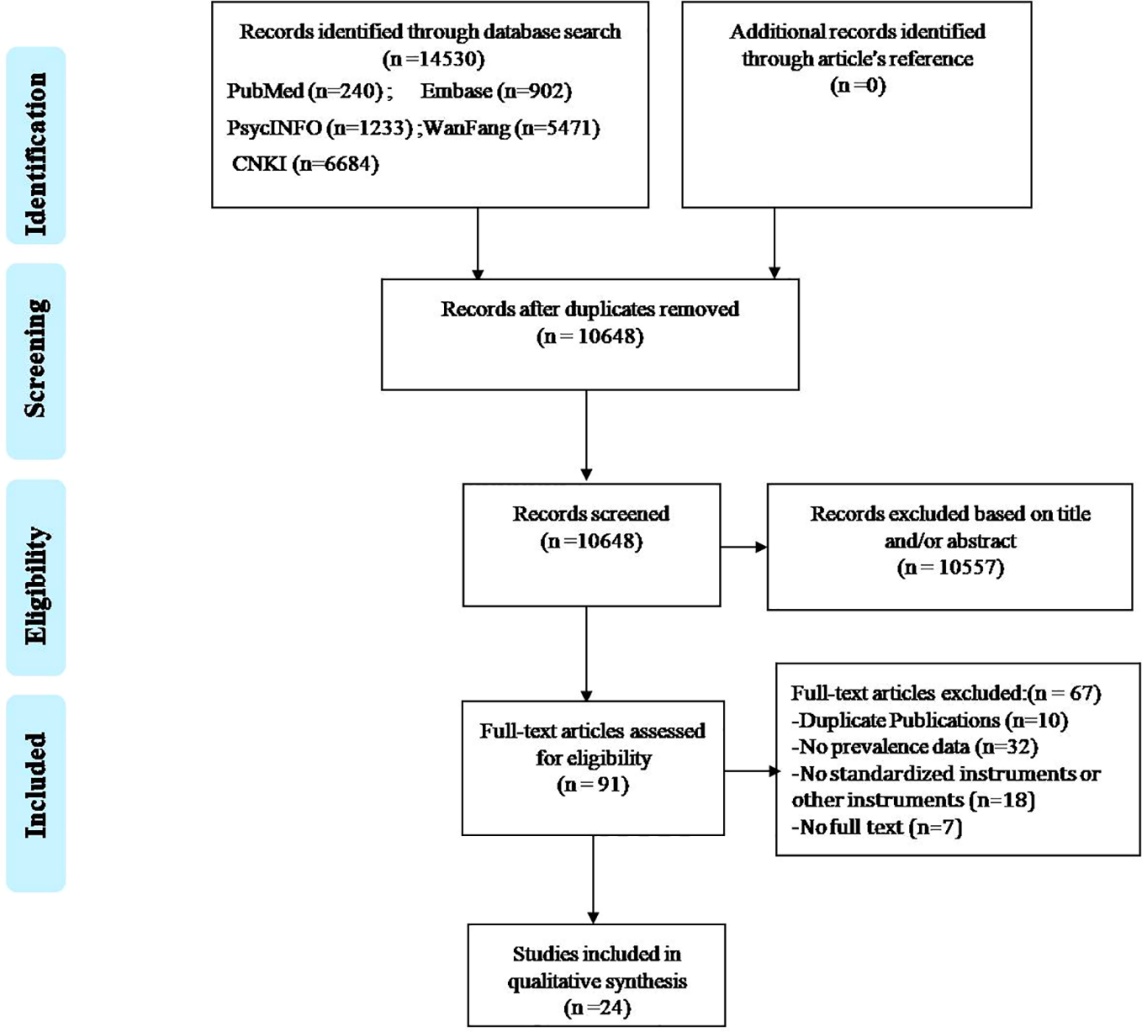
PRISMA flowchart.

The process of the literature search is shown in **Figure 1**. Two investigators systematically and independently searched PubMed, EMBASE, PsycINFO, WanFang, and Chinese National Knowledge Infrastructure from their inception date to Sep 16, 2017 with the following search terms: (“China” OR “Chinese” OR “Hong Kong” OR “Taiwan” OR “Macao”) AND (“insomnia” OR “sleep symptom” OR “sleep disorder” OR “sleep quality” OR “sleep disturbance” OR “sleep problem” OR “sleep time” OR “sleep duration” OR “sleep habit” OR “sleep pattern”) AND (“Hypertension” OR “hypertens*” OR “blood pressure” OR “high blood pressure”). The reference lists of the identified papers were also searched for any additional studies that may have been missed.

### Study Selection

According to the Preferred Reporting Items for Systematic Reviews and Meta-analyses (PRISMA) recommendation (33), the inclusion criteria based on the PICOS acronym were used in this meta-analysis: Participants (P): patients with hypertension. The diagnosis of hypertension was established according to international diagnostic criteria, such as the European Society of Hypertension (ESH) and the European Society of Cardiology (ESC) Guidelines on hypertension (34), or local diagnostic criteria in China, such as the Chinese guidelines for the management of hypertension (35); Intervention (I): not applicable; Comparison (C): healthy subjects in case control and cohort studies; and not applicable in cross-sectional studies without control groups, such as epidemiological surveys; Outcomes (O): reported data of poor sleep quality as defined by PSQI cutoff values; and Study design (S): cross-sectional or cohort studies conducted in mainland China, Hong Kong, Macao, and Taiwan (only baseline data were extracted in cohort studies). Two investigators (LiLi and J-XC) screened the titles, abstracts, and full-texts of the initial search results independently. Any discrepancies that emerged in these procedures were discussed and resolved by involving a third investigator (LuLi).

### Quality Evaluation

Two investigators (LiL and J-XC) independently assessed the methodological quality of the studies using a quality assessment tool consisting of eight items in terms of sampling, measurement, and analysis (**Supplementary Table 1**). The item scores range between 0 and 8, with a score of 7–8 as high quality, 4–6 as moderate quality, and 0–3 as low quality (36). Disagreements between the two investigators were resolved by discussing with a third investigator (LuL).

### Data Extraction

Data were independently extracted by two investigators (LiL and J-XC) and checked by a third investigator (LuL). The following information was extracted and tabulated: study site and time, geographic region, study location, sampling method, mean age, proportion of males, sample size, type of hypertension and cut-off values of instrument on sleep quality, the prevalence of poor sleep quality, and quality assessment. The hospital population refers to the studies that were conducted in hospitals in which participants received treatments, while community population refers to studies of participants with hypertension who lived in the community and received treatments in community clinics or outpatient clinics of general hospitals. In China, maintenance treatments of physical diseases are mainly provided by community clinics or outpatient clinics attached to general hospitals. Hospital- or community-based studies were classified based on the respective study-defined criteria.

### Statistical Analyses

The Comprehensive Meta-Analysis Program, Version 2 (Biostat Inc., Englewood, New Jersey, USA) was used to perform the data analysis. Due to different demographic characteristics and sampling methods, data on poor sleep quality were combined using the random-effects model; prevalence and odds ratio (OR) with 95% confidence intervals (CIs) were indicated as effect size. The I^2^ statistic and Cochran’s Q test were used to evaluate heterogeneity between studies, with I^2^ values greater than 50% indicating great heterogeneity (37). In order to examine the moderating effects of associated factors on the results, the subgroup analyses were conducted based on the following categorical variables: community-based studies, publication language, geographic region, age, survey year, sample size, sampling method, and PSQI cut-offs. In addition, meta-regression analyses were conducted to examine the moderating effects of continuous variables, such as age, year of survey, proportion of males, and the sample sizes. Only studies reporting the above-mentioned data were included in subgroup or meta-regression analyses. Median splitting methods of continuous variables, such as age, survey year, and sample size, were used in the subgroup analyses. If the results of subgroup and meta-regression analyses were not consistent, the latter was preferred. Sensitivity analysis was conducted by removing each study individually to evaluate the consistency of the results. The funnel plot and Egger’s tests were used to assess publication bias. All analyses were two-tailed, with alpha set at 0.05.

## Result

### Search Results, Studies Characteristics, and Quality Assessment

**Figure 1** shows the flow chart of the search and selection process. Finally, 24 studies met the inclusion criteria. The PSQI-Chinese version was used in all studies. **Table 1** shows the basic characteristics of the included studies which covered 17 provinces and 2 municipalities in mainland China. All studies were rated as “moderate quality” or “high quality”; the mean score of the quality assessment was 5, ranging from 4 to 7.

**Table 1:** Characteristics of the studies included in the meta-analysis.

No.	First author	Study area (Region)	Year of survey	Study location	Sampling method	Sample size	Mean age	Proportion of male (%)	Type of hypertension	Scale score	Cut-off score	Rate of hypertension (%)	Quality assessment
1	Fang et al. (38)	Shanghai (S)	2014	Community	C	1,606	72 (65–80)	47.14	NR	7.61 ± 3.23	>7	43.2	5
2	Zhang et al. (20)	Hubei (S)	2014	Hospital	Conv	70	43.4 ± 10.4	60	Essential/Secondary	9.73 ± 3.47	>7	85.7	6
3	Du et al. (39)	Jilin (N)	2015–2016	Community	Conv	208	64.6 ± 6.5	51.92	NR	6.86 ± 2.28	>7	43.75	5
4	Hu et al. (40)	Hunan (S)	2013–2014	Hospital	R	610	67.5 ± 7.1	55.74	Essential	NR	>10	47.4	6
5	Mao et al. (23)	Yunnan (S)	2015	Community	C	793	68.0 ± 5.6	45.4	NR	5.7 ± 2.9	>7	14.9	5
6	Xiao et al. (41)	Guangdong (S)	2013–2016	Hospital	Cons	176	68.0 ± 4.2	NR	Essential	NR	>7	55.68	5
7	Huang (42)	Fujian (S)	2013–2014	Hospital	Cons	256	58.5 (30–80)	55.86	Essential	NR	>6	32.8	5
8	Liu et al. (7)	Liaoning (N)	2012–2013	Community	M, R	4,800	52.1 ± 14.1	50.72	NR	5.01 ± 2.71	>5	36.02	7
9	Ma et al. (43)	Shanxi (N)	2014	Hospital	Cons	135	49 ± 6.4	100	Essential	NR	>5	71.1	5
10	Zheng et al. (44)	Fujian (S)	2013–2014	Community	Conv	729	60.3 ± 9.2	54.32	Essential	5.39 ± 2.77	>7	28.67	4
11	Yu et al. (45)	Chongqing (S)	2013–2014	Hospital	Cons	378	54.7 ± 11.8	51.06	Essential	3.65 ± 2.94	>5	56.35	6
12	Wei et al. (46)	Guangxi (S)	2009–2013	Hospital	Cons	186	70.6 ± 9.7	54.84	Essential	NR	>7	48.39	5
13	Zhang et al. (47)	Zhejiang (S)	2010–2012	Community	Cons	97	62.7 (50–78)	48.45	NR	NR	>6	80.4	4
14	Zhu et al. (48)	Shanghai (S)	2012	Community	R	457	64.7 ± 9.60	53.61	Essential	NR	>6	65.34	5
15	Fang et al. (49)	Hunan (S)	NR	Community	R	145	75.3 ± 12.9	55.17	Essential	8.98 ± 3.36	>7	45.67	6
16	Wang et al. (50)	Guangdong (S)	2012	Hospital	Cons	75	50.0 ± 8.7	57.33	NR	7.80 ± 3.95	>7	44	4
17	Wen et al. (51)	Shanxi (N)	2012–2013	Hospital	Cons	268	35–75	45.15	Essential	NR	>7	50	5
18	Luo et al. (52)	Shanghai (S)	NR	Community	C	629	NR	NR	NR	NR	>5	44.67	6
19	Dong et al. (53)	Anhui (S)	2009	Community	C, R	1,110	69.1 ± 6.87	51.89	NR	7.65 ± 3.91	>7	42.7	5
54	Cheng et al. (54)	Guangdong (S)	NR	Community	Cons	122	67.9 ± 6.1	54.92	Essential	8.34 ± 3.81	>7	63.9	5
21	Xie et al. (55)	Xinjiang (N)	2008–2009	Hospital	Cons	760	56.3 ± 16.6	57.5	NR	8.42 ± 3.08	>7	62.9	5
22	Zhang et al. (56)	Guangdong (S)	2007–2008	Hospital	Cons	100	74.0 ± 6.3	52	NR	9.54 ± 3.00	>7	76	5
23	Sun et al. (57)	NR	2005–2006	Hospital	Cons	139	54.6 ± 18.7	63.31	Essential	10.96 ± 2.33	>7	69.8	6
24	Zhang et al. (58)	Gansu (N)	NR	Hospital	Cons	71	52.1 ± 12.7	64.79	NR	10.86 ± 5.10	>10	56.34	5

### Prevalence of Poor Sleep Quality

**Figure 2:**
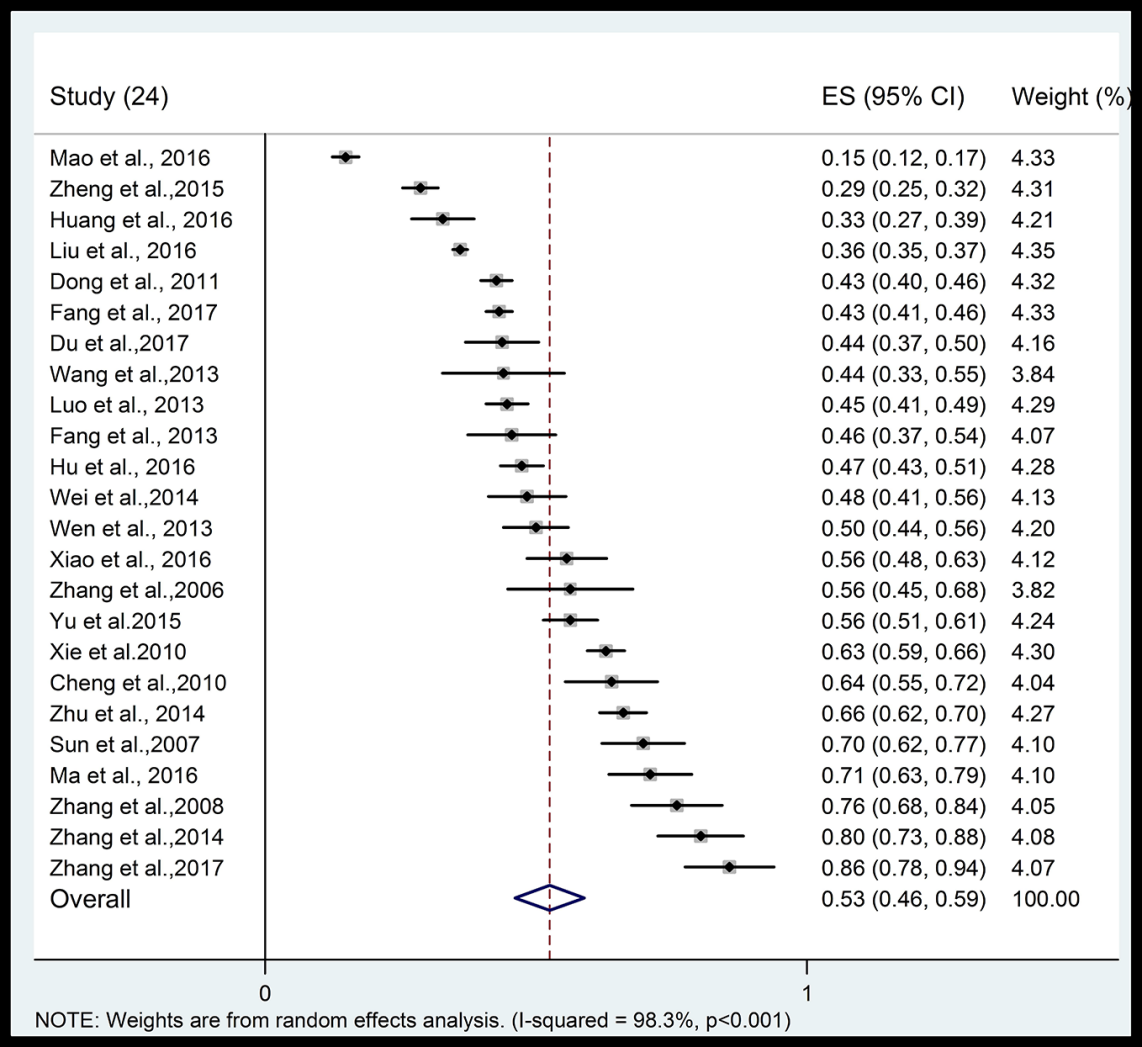
Forest plot of prevalence of poor sleep quality in hypertensive patients.

**Figure 2** shows the forest plot of the prevalence of poor sleep quality. The pooled prevalence of poor sleep quality in 24 studies with 13,920 hypertensive patients was 52.5% (95% CI: 46.1–58.9%) with significant heterogeneity (I^2^: 98.3%), ranging from 14.9 to 85.7%. The pooled prevalence of poor sleep quality in 6 studies with 5,610 healthy controls was 32.5% (95% CI: 19.0–49.7%) with significant heterogeneity (I^2^: 98.3%). **Supplemental Figure 1** indicates that the hypertensive patients were more likely to have poor sleep quality than healthy controls (OR: 2.66, 95% CI: 1.80–3.93) with significant heterogeneity (I^2^: 89.3%) from the six case-control studies with available data.

### Subgroup and Meta-Regression Analyses

The results of the subgroup analyses are shown in **Table 2**. There was no significant difference in the prevalence of poor sleep quality between different geographical regions. Hypertensive patients in hospitals were more likely to suffer from poor sleep quality than those in the community (58.2 *vs.* 45.1%, P = 0.012). The prevalence of poor sleep quality was higher in studies published in Chinese than English (53.6 *vs.* 40.0%, P = 0.016). Studies with smaller sample size reported a higher rate of poor sleep quality (62.3 *vs.* 42.9%, P = 0.001), while those using convenience or consecutive sampling reported a higher rate of poor sleep quality (41.5 *vs.* 58.2%, P = 0.005). Meta-regression analyses revealed that the proportion of poor sleep quality was higher in the studies with smaller sample size (β = −0.00012, p < 0.001) and was negatively associated with survey year (β = −0.114, P < 0.001). The poor sleep quality was more common in male patients (β = 3.46, P < 0.001) and in older patients (β = 0.00608, P < 0.001).

**Table 2:** Subgroup analyses.

Subgroups	Categories (Number of studies)	Proportion (%)	95% CI(%)	Events	Sample size	*I^2^* (%)	Q (P)
**Community**	Yes (11)	45.1	37.7–52.8	4,119	10,696	97.8	**6.32 (0.012)**
	No (13)	58.2	51.4–64.6	1,788	3,224	92.2	
**Geographical region**	North (6)	53.3	40.0–66.2	2,567	6,242	98.0	0.09 (0.76)
	South (17)	51.0	43.6–58.3	3,243	7,539	97.1	
**Publication language**	Chinese (22)	53.6	46.7–60.4	3,899	8,491	97.0	**5.80 (0.016)**
	English (2)	40.0	32.0–53.8	2,009	5,429	94.2	
**Age group^a^**	≥63.7 (11)	57.1	46.5–67.0	3,117	7,510	97.8	1.34 (0.24)
	<63.7 (11)	48.9	39.9–57.9	2,373	5,513	97.3	
**Survey year^a^**	2013–2017 (10)	46.9	36.6–57.4	1,952	4,961	97.7	2.28 (0.13)
	2007–2012 (10)	57.9	48.2–67.0	3,488	7,992	97.8	
**Sample size^a^**	≥232 (12)	42.9	36.0–50.2	5,002	12,396	98.1	**11.8 (0.001)**
	<232 (12)	62.3	54.1–69.9	903	1,524	89.7	
**Sampling method**	Probability (8)	41.5	33.9–49.5	3,950	10,150	98.0	**8.03 (0.005)**
	Non-probability (16)	58.2	49.8–66.1	1,955	3,770	95.6	
**Cut-off of CPSQI**	>5 (4)	51.7	39.2–63.8	2,319	5,942	50.4	0.52 (0.91)
	>6 (3)	60.5	33.8–82.2	461	810	97.5	
	>7 (15)	51.1	42.6–59.5	2,796	6,487	97.7	
	>10 (2)	50.1	42.1–58.1	329	681	97.3	

Bolded values: P < 0.05; Q: Cochran’s Q;

a: Continuous variables, such as age, survey year and sample size, were dichotomized using median splitting methods in the subgroup analyses. CPSQI: Chinese version of the Pittsburgh Sleep Quality Index; Probability sampling method: cluster sampling; multistage sampling; random sampling; stratified sampling; Non-probability sampling method; convenience and consecutive sampling.

### Publication Bias

**Figure 3:**
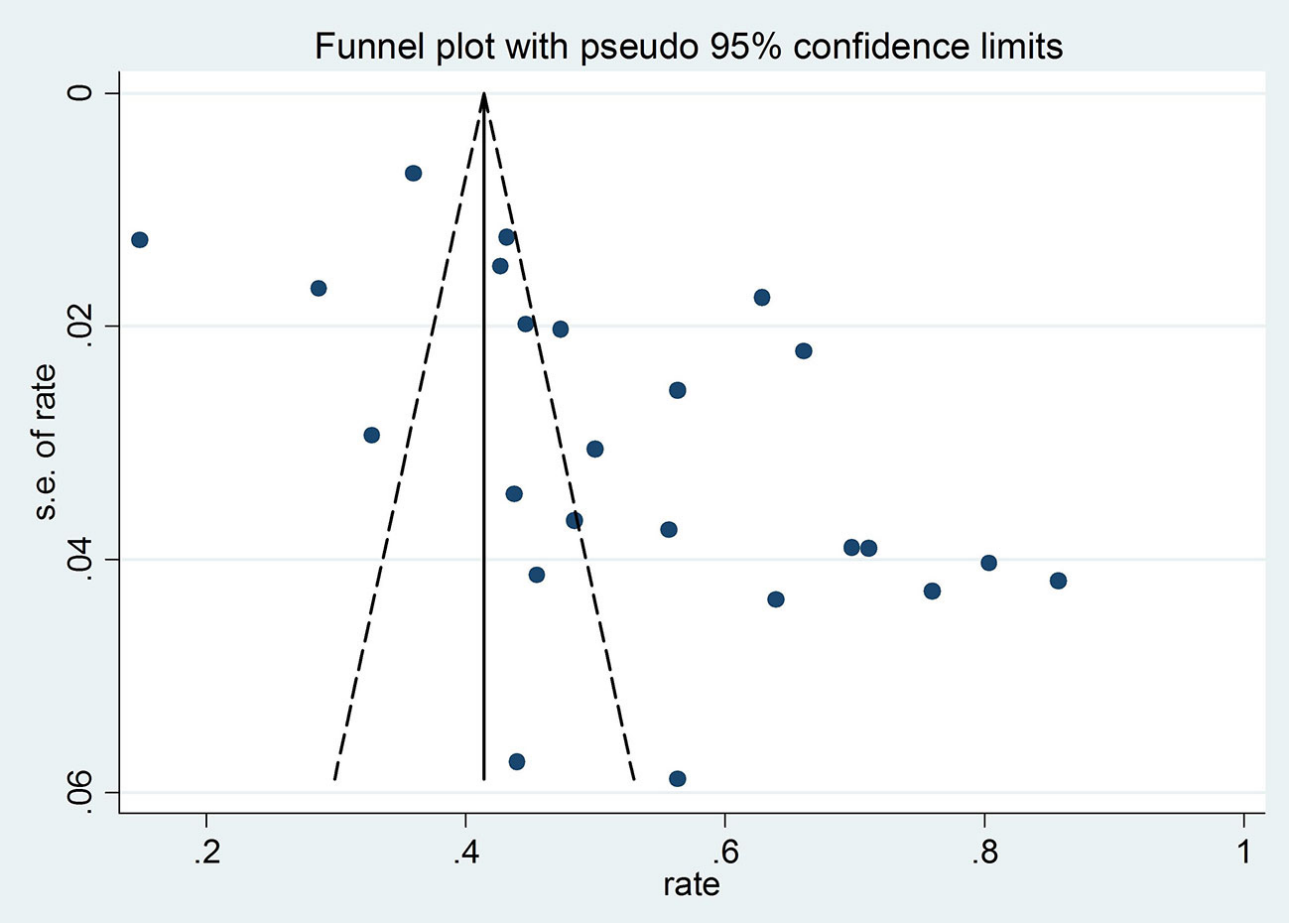
Funnel plot of publication bias for 24 studies with available data on prevalence of poor sleep quality.

Visual funnel plot and the Egger’s tests (t = 6.18, P < 0.001) both indicated significant publication bias (**Figure 3**).

### Sensitivity Analysis

When studies were excluded one by one, the recalculated results did not change significantly. Therefore, no individual study significantly influenced the primary results.

## Discussion

This was the first meta-analysis of the prevalence of poor sleep quality in hypertensive patients in China. We found that more than half of the patients with hypertension had poor sleep quality, which is around two times higher than in healthy controls (OR: 2.66). There were approximately 325 million hypertensive patients in China, which translates to around 170.6 million hypertensive patients with poor sleep quality based on a prevalence of 52.5% (26). Several factors may be associated with the increased rate of poor sleep quality in hypertensive patients. First, hypertension symptoms, such as pain and headache (5), are associated with sleep problems, such as poor sleep quality (59). Several studies found that poor sleep quality in hypertension could offset the effects of blood pressure control (60). In addition, poor sleep quality is usually significantly associated with impaired physical functioning and poor mental health in patients with hypertension (21); therefore, improving sleep quality might be beneficial in improving both mental health and hypertension. Second, hormones involved in the regulation of the sleep/wake cycle could reduce systolic blood pressure and diastolic blood pressure, and enhance nocturnal systolic and diastolic blood pressure dipping in hypertensive patients (61). Thus, long-term poor sleep quality could disturb the rhythm of body clock and influence catecholamine secretion, which may result in high blood pressure. Third, there is a close association between sleep and high blood pressure since both are linked to the activities of hypothalamo-pituitary-adrenal (HPA) axis (62, 63). For example, untreated obstructive sleep apnea (OSA) is significantly related to the development of hypertension; OSA can result in intermittent hypoxemia and cause oxidative stress, which is associated with increased sympathetic activity by the HPA axis, and hence elevated blood pressure (64).

The subgroup analyses found that hypertensive patients in hospitals had a higher risk of poor sleep quality than those in the community (58.2 *vs.* 45.1%, P = 0.012). It is likely that patients in hospitals could have more severe hypertension and more comorbidities, which could increase the risk of poor sleep quality. Similar to other studies (65), there was a positive association between poor sleep quality and older age. On the one hand, both sleep duration and quality usually decrease with age. On the other hand, older adults usually have low levels of outdoor activities and high rates of physical and psychological problems, such as diabetes, dementia, respiratory disease, and depression, all of which could increase the risk of poor sleep quality (66, 67).

Unlike previous findings (7, 22, 68), we found that male patients were more likely to have poor sleep quality (β = 3.46, P < 0.001), which could be attributed to gender difference in the use of sleep promoting medications in China although medication use was not analyzed in this meta-analysis due to inadequate information. Compared with males, Chinese females with sleep problems are more likely to accept sleep promoting medications (68), which may improve sleep quality.

Studies with small sample size reported a higher rate of poor sleep quality (β = −0.00012, p < 0.001), while those using convenience or consecutive sampling also reported a higher rate of poor sleep quality. We assumed that studies with small sample size and those using convenience or consecutive sampling may have relatively unstable results (69). We also found a negative association between poor sleep quality rate and year of survey (β = −0.114, P < 0.001). This is perhaps due to remarkable increases in coverage and utilization of healthcare resources seen in recent years in China (70), which could reduce the risk of poor sleep quality. The funnel plot and Egger’s tests indicated that publication bias exist, but the exact reasons are unclear. The possible reasons may be that studies with a small sample size and/or those with a lower prevalence of poor sleep quality were less likely to be published by academic journals, which could result in publication bias (71). In addition, the prevalence of poor sleep quality in studies published in Chinese appeared to be higher than those published in English, which is probably due to the very small number of studies in the English group (n = 2).

The strength of this meta-analysis includes the moderate-high quality of the included studies that were conducted across broad regions of China. However, several limitations should be considered. First, certain factors related to sleep quality in hypertensive patients, such as education level, physical exercise, anti-hypertensive treatments, and duration of hypertension, were not examined due to inadequate data. In addition, poor sleep quality had a bidirectional association with psychiatric disorders (14, 15). However, psychiatric comorbidities in hypertensive patients were not reported in most of the included studies, therefore, their moderating effects on the results could not be examined. Second, similar to other meta-analyses of epidemiology (72–74), there was significant heterogeneity of prevalence estimate across studies probably due to the discrepancy in study year, psychiatric and somatic comorbidities, sampling methods, and demographic characteristics of patients (75). The substantial heterogeneity in the subgroup analyses is usually unavoidable in meta-analyses of observational and epidemiological surveys (75–78) even if the subgroup analyses are conducted. Third, several studies had a small sample size and/or used non-random sampling, which probably had contributed to the publication bias (79, 80). Fourth, the potential confounding effects between moderating variables could not be controlled for because the statistical programs used in this study could only perform univariate analyses. Finally, the PSQI is the only standardized scale on subjective sleep quality available in China, which therefore could reduce the possibility of missing relevant studies in this meta-analysis. However, those using objective measures (e.g., polysomnography) on sleep quality were not included.

In conclusion, more than half of the Chinese patients with hypertension in this meta-analysis suffered from poor sleep quality which was significantly associated with male gender and older age. Considering the negative impact of sleep quality, appropriate strategies for the screening, prevention, and treatment of poor sleep quality in hypertensive patients should be developed.

## Data Availability Statement

The raw data supporting the conclusions of this article will be made available by the authors, without undue reservation, to any qualified researcher.

## Author Contributions

Study design: LiL, Y-TX. Data collection, analysis, and interpretation: LiL, LuL, J-XC, LX. Drafting of the manuscript: LiL, Y-TX. Critical revision of the manuscript: CN, GU.

## Funding

The study was supported by the National Science and Technology Major Project for investigational new drug (2018ZX09201-014), the Beijing Municipal Science & Technology Commission (No. Z181100001518005), the University of Macau (MYRG2019-00066-FHS) and Science and Technology Plan Project of Guangdong Province (No.2019B030316001).

## Conflict of Interest

The authors declare that the research was conducted in the absence of any commercial or financial relationships that could be construed as a potential conflict of interest.
