# Supplementary figures and images for: Prevalence of Poor Sleep Quality in Patients With Hypertension in China: A Meta-analysis of Comparative Studies and Epidemiological Surveys
Source: Front Psychiatry. 2020 Jun 30;11:591. doi: 10.3389/fpsyt.2020.00591 (PMC7338685; doi:10.3389/fpsyt.2020.00591)

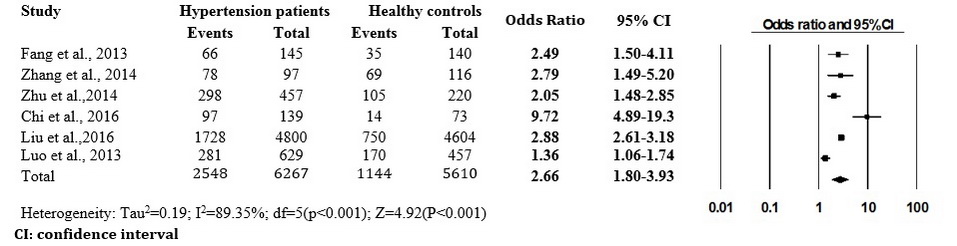

Supplement: Supplementary file 1 [file Image_1.jpeg]
