# Supplementary material for: Prevalence of Poor Sleep Quality in Patients With Hypertension in China: A Meta-analysis of Comparative Studies and Epidemiological Surveys
Source: Front Psychiatry. 2020 Jun 30;11:591. doi: 10.3389/fpsyt.2020.00591 (PMC7338685; doi:10.3389/fpsyt.2020.00591)
Supplement: Supplementary file 2 [file Table_1.docx]

**Supplemental table 1** The items of quality assessment

| 1 | Is the target population clearly defined? |
| --- | --- |
| 2 | Was either of the following ascertainment methods used [must be one or the other]? (1) probability sampling, or (2) entire population surveyed |
| 3 | Is the response rate >70% |
| 4 | Are non-responders clearly described? |
| 5 | Is the sample representative of the target population? |
| 6 | Were data collection methods standardized? |
| 7 | Were validated criteria used to assess for the presence/absence of disease? |
| 8 | Are the estimates of prevalence given with confidence intervals and in detail by subgroup (if applicable)? |
